# LACE Index to Predict the High Risk of 30-Day Readmission: A Systematic Review and Meta-Analysis

**DOI:** 10.3390/jpm12040545

**Published:** 2022-03-30

**Authors:** Vasuki Rajaguru, Whiejong Han, Tae Hyun Kim, Jaeyong Shin, Sang Gyu Lee

**Affiliations:** 1 Department of Healthcare Management, Graduate School of Public Health, Yonsei University, Seoul 03722, Korea; vasuki@yuhs.ac (V.R.); hanw@yuhs.ac (W.H.); thkim@yuhs.ac (T.H.K.); 2 Department of Preventive Medicine, College of Medicine, Yonsei University, Seoul 03722, Korea; drshin@yuhs.ac; 3 Institute of Health Services Research, Yonsei University, Seoul 03722, Korea

**Keywords:** 30-day readmission, hospital readmissions, quality improvement, relative risk

## Abstract

The LACE index accounts for: Length of stay (L), Acuity of admission (A), Comorbidities (C), and recent Emergency department use (E). This study aimed to explore the LACE index to predict the high risk of 30-day readmission in patients with diverse disease conditions by an updated systematic review. A systematic review carried out by electronic databases from 2011–2021. The studies included a LACE index score for 30-day of readmission and patients with all types of diseases and were published in the English language. The meta-analysis was performed by using a random-effects model with a 95% confidence interval. Of 3300 records, a total of 16 studies met the inclusion criteria. The country of publication was primarily the USA (*n* = 7) and study designs were retrospective and perspective cohorts. The average mean age was 64 years. The C-statistics was 0.55 to 0.81. The pooled random effects of relative risk readmission were overall (RR, 0.20; 95% CI, 0.12–0.34) and it was favorable. The subgroup analysis of the opted disease-based relative risk of readmissions of all causes, cardiovascular and pulmonary diseases, and neurological diseases were consistent and statistically significant at *p* < 0.001 level. Current evidence of this review suggested that incorporating a high-risk LACE index showed favorable to risk prediction and could be applied to predict 30-day readmission with chronic conditions. Future study would be planned to predict the high risk of 30-day readmission in acute clinical care for utility, and applicability of promising LACE index in South Korean hospitals.

## 1. Introduction

Hospital readmissions, especially unplanned ones, are costly for the healthcare industry [1]. Readmission frequency is used to judge hospital quality as 30 days of unplanned readmission indicates the initial intervention was unsuccessful [2]. The Centers for Medicare and Medicaid Services (CMS) reported annual medical expenditures of $17 billion as a result of hospital readmissions. CMS described chronic conditions with a high risk of frequent hospitalization as part of the 2010 Hospital Readmission Reduction Program (HRRP) [3].

The readmission rate metric was first developed in the United States (US) for quality improvement and cost reduction and is being used in several countries such as Canada [4], Australia [5], and the United Kingdom [6]. Policies such as the Affordable Care Act’s (ACA) Hospital Readmission Reduction Program (HRRP) have attempted to improve quality by penalizing 30-day readmission rates above the national standard in the US [4,5,6,7], Continuous quality improvement in local healthcare systems can lower readmission rates and cut costs, boosting the global economy. Beyond these assuagements, more sensitive methods and algorithms are needed to predict which patients are at risk of readmission before they are discharged.

There are several tools and scoring patterns that have been reported to measure or predict the risk of readmissions [8,9,10]. The LACE index is one of the most commonly used indices in the Canada [9,10,11], and US [12,13,14,15,16,17,18]. The LACE index was first developed by van Walraven et al. [9] to predict the risk of unplanned readmission or death within 30 days after hospital discharge in medical and surgical patients. The model was derived and validated based on administrative data with a C-statistic of 0.68. The model includes the length of hospitalization stay (L), acuity of the admission (A), comorbidities of patients (C), and the number of emergency department visits in the six months before admission (E). Scores ranging from “0” to “19” and greater than ten are considered high risk for 30-day readmission [9]. The higher scores indicate a high risk of readmission. This tool is widely used primarily because of its simplicity makes it usable in day-to-day clinical practice [9,10,11,12,13,14,15,16,17,18].

To this end LACE index was utilized in various settings including The Canadian Institute for Health Information (CIHI) evaluated the quality of care by suggesting 30-day unplanned readmissions in acute care that considered patient, hospital, and community factors [4,10]. The UK used the Emergency Readmission to Hospital within 28 Days of Discharge to monitor readmissions [19]. In Australia, the Ministry of Health of the Western Australia provincial government used 30-day unplanned readmissions for surgical events and all cause admissions as a health service quality metric [20,21,22]. However, there is a question as to whether it is appropriate to apply the indicator in other regions across a range of settings and populations.

Multiple studies have been conducted to address the unplanned 30-day readmission after discharge from the hospital, which becomes an indicator of the quality of the healthcare system in South Korea [23,24] and also stands to benefit from a reduction in hospital readmissions. However, the readmission rate is an index that can be calculated using administrative data along with the mortality rate. As a result, discussion around the appropriate use of the LACE index has been emphasized. The review of risk prediction for 30-day readmissions in a health care facility is a very important concern for economic as well as quality considerations. Therefore, this study aimed to review the scientific articles related to the LACE index systematically and undertake a meta-analysis of available data relevant to predict 30-day readmission.

## 2. Materials and Methods

This systematic review was registered with the International Prospective Register of Systematic Reviews (PROSPERO, CRD442021284055). We followed the Preferred Reporting Items for Systematic Reviews and Meta-analyses (PRISMA) reporting guideline [25,26] and check list (Supplementary Materials Table S1). This systematic review will be utilized and reported in compliance with our forthcoming research.

### 2.1. Eligibility Criteria

Studies were included based on the following criteria: (1) articles published from 2011 to 2021 (2) study design clearly stated 30-day readmission risk prediction with the LACE index (3) related content patients or they investigated the risk of inappropriate 30-day admission and predictive model based on the LACE score or measurements were performed with a valid standard or protocol. Studies were excluded if (1) the researchers did not have access to full-text (2) the methodologic quality of the studies was too low and non-relevant content of 30-day readmissions, and (3) conference, case reports, and review articles.

### 2.2. Search Strategy and Data Sources

The literature was searched in the following electronic databases from October 2021 to December 2021, including PubMed, Embase, Scopus and Web of Science, and Cochrane library databases. The electronic search strategy was reviewed according to the Peer Review of Electronic Search Strategies, to enhance the quality. The reference lists of the included studies were also hand-searched. No restriction regarding publication status or design was applied. The complete search strategy for the electronic database is provided in an example Supplementary Materials Table S2.

### 2.3. Study Selection and Data Extraction

The retrieved records were imported into the EndNote Ver.8 (Clarivate Analytics, Philadelphia, PA, USA), and then the duplicates were removed. The collected articles were restructured and numbered by order. Two independent researchers (V.R.; J.S.) screened the titles, abstracts, and full texts of potentially eligible studies. Data were extracted in PRISMA-ScR Checklist. The research team conducted the appraisal collaboratively and full-text versions were evaluated to determine inclusion and exclusion by two reviewers (J.S.; W.H.). The team supervisors (T.H.K; S.G.L) examined the reviewed articles to resolve any disagreements through several meetings. The reference lists of relevant articles were also examined for other potential eligibility for selection. It was followed by data extraction, including author names, publication year, sample size, study design, study period (years), primary outcome, time of readmission, measurement, type of readmissions (through an emergency visit, inpatient department, or both, transfer from the other hospitals), and findings. We then assessed for data evaluating study design, methodology, and reporting.

### 2.4. Quality Assessment

All the potentially relevant articles were extracted and reviewed in full by the same two authors for methodological validity before inclusion in the review using standardized methodological quality using the Cochrane Risk of Bias Tool for Risk Of Bias In Non-randomized Studies (ACROBAT-NRSI) [27]. Disagreement on article consent was resolved by discussion between the third and fourth authors.

### 2.5. Data Synthesis

All the selected studies are divided into two groups, including 30-day readmission and no readmission. The total population considered as 30-day readmissions were ‘Yes’ (intervention group) and ‘No’ (control group). We performed the random-effects model meta-analyses to estimate the pooled risk ratios, and the 95% confidence intervals and heterogeneity of the studies outcomes were assessed using I^2^, where no covariates are obvious contenders to explain the heterogeneity, random-effects meta-analysis is appropriate [28]. We also examined the 30-day readmission among those studies considering an undifferentiated high-risk chronic disease population.

## 3. Results

### 3.1. Study Selection

**Figure 1:**
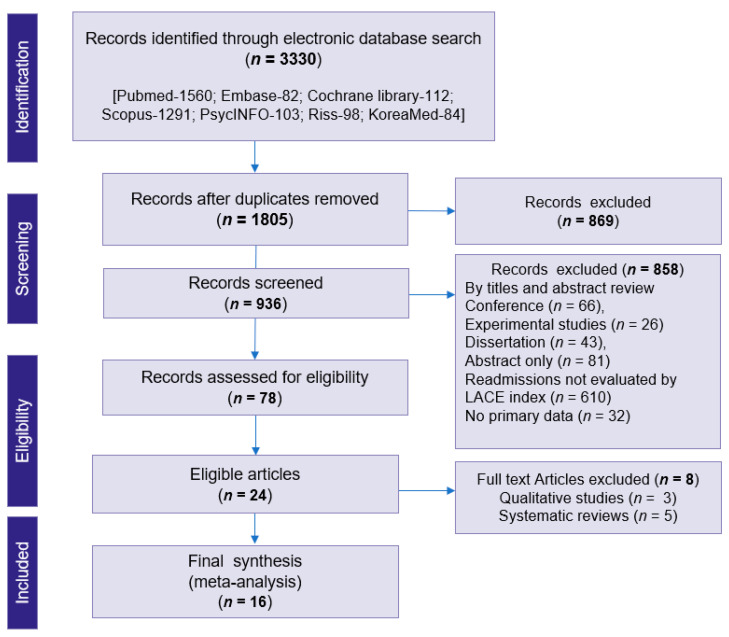
Flow diagram of studies included in the review based on the Preferred Reporting Items for Systematic Review and Meta-Analysis Guidelines.

The electronic search yielded 3330 articles and 936 were selected based on title and abstract screening after removing the duplicates. From those, 657 articles were assessed for eligibility. 634 articles did not meet the eligibility criteria or there was non-availability of full-text access, and they were thus excluded. Of these, 16 studies were selected for an in-depth review process. The selection process of the articles is given in the flow diagram in Figure 1.

### 3.2. Characteristics of the Review Articles

There were sixteen articles included. Table 1 summarizes the characteristics of the reviewed studies. Most of the studies were conducted in the USA (*n* = 7), Canada (*n* = 2), Australia (*n* = 3), Singapore (*n* = 2) and UK (*n* = 2). Most of studies were retrospective cohort design (*n* = 14), prospective (*n* = 1) and Survey (*n* = 1). The target population were cardiovascular diseases (*n* = 4), respiratory diseases [Chronic obstructive pulmonary disease (COPD) and pneumonia] (*n* = 3), all-cause admissions (*n* = 7), and neurological conditions including surgery (*n* = 2). The study setting and data resources were university hospitals (*n* = 9) tertiary and referral hospitals (*n* = 3), public hospitals (*n* = 2), and administrative healthcare database (*n* = 2). Data collection period ranged from one year (hospital data) to 10 years (National Health care data), which was an average period between 2003 and 2018. All the studies included adult patients aged ≥18 years, and the mean age ranged from 55–72 years have been reported. The measurable variables are baseline data and score of LACE index (*n* = 11) only and a combination of hospital score or LACE+ index (*n* = 6). Most of the studies analyzed the prediction model of 30-day readmission by using logistic regression analysis. The prediction model outcomes included all-cause admissions (*n* = 7), cardiovascular, and pulmonary diseases (*n* = 7), and neurological conditions (*n* = 2) including surgery. (Table 1).

### 3.3. Meta-Analysis

Table 2 summarizes the baseline characteristics of all the selected studies and data were utilized for meta-analysis. The meta-analysis findings for the C-statistics are practically more or less the same in all the LACE index implementation strategies. The risk prediction model was developed by using the cohort data based on study populations, categorized: 30-day readmissions ‘Yes’ or ‘No’ (Table 2).

**Figure 2:**
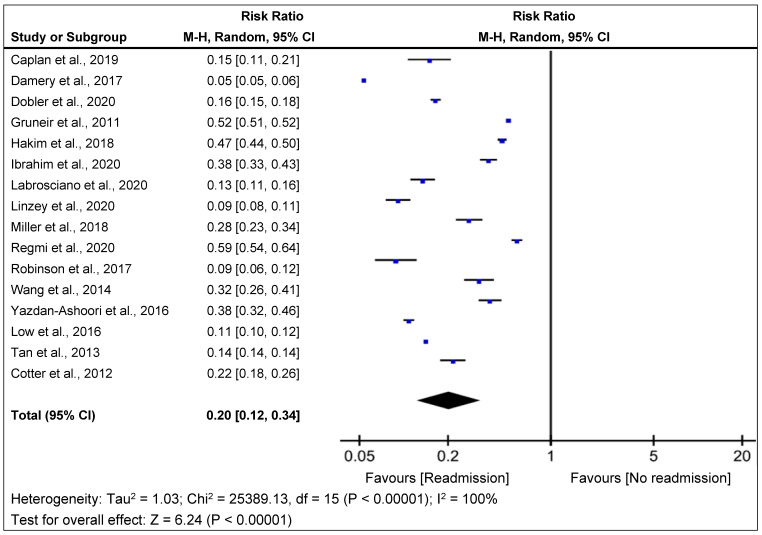
Forest plot of LACE Risk Ratio for 30-day hospital admissions with LACE index in overall studies [12,13,14,15,16,17,18,19,20,21,22,29,30,31]. Boxes indicate the risk ratios (RRs); whiskers, 95% CIs; diamonds, pooled RR of readmission; vertical straight lines, overall pooled RR of 1.00. M-H = Mantel-Haenszel; CI = confidence interval.

**Figure 3:**
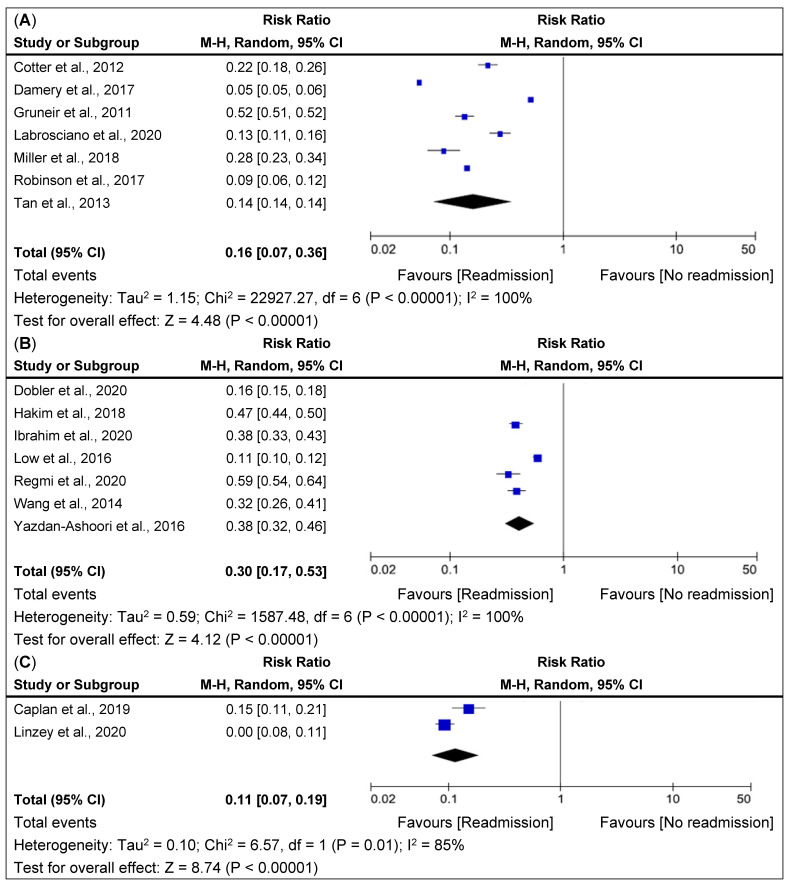
Forest plot of LACE Risk Ratio for 30-day hospital admissions with LACE index in by different disease conditions based on the selected studies. (**A**). All cause readmissions [10,15,16,19,22,30,31], (**B**), Cardiovascular and Pulmonary conditions [11,13,17,18,20,21,29]; (**C**) Neurological conditions [12,14]. Boxes indicate risk ratios (RRs); whiskers, 95% CIs; diamonds, pooled RR of readmission; vertical straight lines, overall pooled RR at 1.00 level. M-H = Man-tel-Haenszel; CI = confidence interval.

The meta-analysis results for each statistic are shown in Figure 2 and Figure 3. The overall pooled relative risk (RR) of readmission within 30-day readmission was 20% (95% CI, 0.13–0.29; *p* < 0.001) (Figure 2). Variability in readmission RR was high (I^2^ = 100%). This finding was consistent with the risk prediction in highly favorable and associated relative risks for 30-day readmissions.

### 3.4. Sub Group Analysis

As several diseases were assessed, we opted to include these in subgroup analysis of readmissions and the results were: all-cause 16% (95% CI, 0.07–0.36; *p* < 0.001), cardiovascular and pulmonary diseases 30% (95% CI, 0.17–0.53; *p* < 0.001) and two neurological diseases 11% (95% CI, 0.07–0.19; *p* < 0.001). Cardiovascular and pulmonary disease related readmissions are showed a higher risk for 30-day readmission than other conditions (Figure 3). The testing model results were associated with the relative risk and favorable to 30-day readmissions.

## 4. Discussion

In this systematic review and meta-analysis study, we performed a scientific literature search with specified inclusion and exclusion criteria and meta-analysis. The primary aim of this study was to assess how well the LACE index was able to predict the high-risk 30-day readmissions by using the cohort data. To calculate the overall estimates of the readmission, we used a randomized effects model o predict the high risk of 30-day readmission by incorporating both readmission and no readmission study variation.

To our knowledge, this systematic review provides the first and in-depth assessment of the LACE index to predict the high risk of 30-day readmissions and includes most of the updated contemporary studies. Our updated literature search identified sixteen studies, most of which are the focused retrospective [12,13,14,16,17,18,19,20,21,22,29,30,31], and prospective [11] cohort and the survey found one in each study design, the source of data included university hospital [11,12,13,14,16,17,19,21,31] public hospital [18,29], tertiary or referral hospital [15,20,22] and administrative health database [10,22]. The data collection was taken in USA [12,13,14,15,16,17,18], Canada [9,10,11] both of which have significant populations, Australia [20,21,22], UK [19,31] and Singapore [29,30] of these South Korea descents.

Numerous studies have been reported on the performance of the LACE index for 30-day readmission risk prediction, some of these have typically been conducted in small patient populations [14,16,29] of adults [30], middle [12,14,16,18,19,20,21], and older aged [10,11,13,15,17,22,31] group. The major disease conditions were included; cardiovascular disease [11,17,18], chronic obstructive pulmonary disease [20,21,29], all-cause [10,15,16,19,30,31] and neurosurgery [26,30]. These variabilities may be due to the varied disease settings including heart failure, craniotomy, neurosurgery, COPD, and pneumonia in the included studies. Interestingly, lung disease patients such as pneumonia and COPD appear to have the greatest risk of readmission, whereas all-cause is relatively low risk as expected. Variability may also be due to the use of LACE+ in addition to standard LACE. Despite a similar name LACE+ is quite different from LACE, having been derived from a logistic regression model [16].

The LACE index was first developed by van Walraven et al. in 2010 [9] to predict the risk of unplanned readmission or death within 30 days after hospital discharge in medical and surgical patients. The model was derived and validated based on administrative data with a C-statistic of 0.68. The model includes the length of hospitalization stay (L), acuity of the admission (A), comorbidities of patients (C), and the number of emergency department visits in the six months before admission (E). All of these variables were frequently cited in all the reviewed studies. However, some studies have been reported LACE index was fair to predict 30-day readmissions and poor prediction in combination of 90 days readmissions and death as well as advanced disease conditions [12,19,29,31]. However, most of the studies found moderate to good discriminative ability. Therefore, interventions might be applied based on the LACE index scores in order to reduce the rate of early readmissions.

Most of the study findings performed the predictive model [11,17,18,19,20,21,22,23,24,25,26,27,28,29,30], the LACE index [10,11,12,13,14,15,16,17,18,19,20,21,22,29,30,31] although validated combined with hospital score [13,16,17], LACE index+ [13] by logistic regression analysis. This study compared the 30-day readmission and no readmission with different disease conditions, the overall pooled relative risk showed favorability in the prediction risk of 30-day readmissions. The variation in LACE score to predict all-cause readmissions [10,15,16,19,30,31] were cardiovascular, pulmonary conditions, and neurological conditions including surgery. Despite the potential heterogeneity of the meta-regression, it showed a significant and incremental effect of “favorable support” on reducing 30-day readmissions.

The discriminative ability of the model, sensitivity, and specificity was calculated in all 16 studies; the C-statistic varied from 0.51 to 0.72 [11,12,13,14,15,16,17,18,19,20,21,22,29,30,31]. Most of the studies performed predictive models at level *p*-value, and the outcomes used the longest follow-up period because intermediate time points were not liable to meta-analysis. Although we did find a statistically significant reduction in readmissions among studies in undifferentiated high-risk chronic diseases, this finding should be interpreted cautiously as there was based on heterogeneity, making interpretation of the composite risk ratio less clear. However, the fact that model accuracy and discriminatory power can be made by testing different predictor variables from routinely collected Electronic medical record (EMR) data may indicate the accuracy of locally relevant clinical or sociodemographic factors.

Our review findings are consistent with the LACE index to predict a high risk of 30-day readmissions. This may have been underestimated in the hospital data used in this review study as the death history of the patients was not added to the data (unless a patient died during index admission). Therefore, it would not be possible to consider the impact of patient mortality in the findings.

This review study has several strengths, including the fact that it is the first systematic review that uses the LACE index in researching 30-day hospital readmissions and the potential utility in facilitating adoption in different countries, including South Korea. LACE index could also act as a decision support tool for physicians that could help them determine whether or not to release an inpatient and to intervene to prevent readmissions.

Future studies within a South Korean population could utilize a retrospective cohort to generate additional variables for regression into the LACE index to create a LACE+ K index with added predictive power and utility in the Korean context.

## 5. Conclusions

Numerous tools and models have been developed to predict hospital readmissions. However, some models are promising and easy to use with adequate discrimination such as the LACE index. It has never been validated in the South Korean population. Therefore, we carried out an updated systematic review and meta-analysis of the use of LACE to predict 30-day readmission with the expectation that it will facilitate the adoption of the algorithm within South Korean hospitals. Our systematic review had a comparable ability to predict the 30-day readmission by using the LACE index for the patients admitted with cardiopulmonary diseases in an acute care setting. It has the advantage of being available to identify the patients at high risk of readmission to receive interventions and potentially avoidable readmission The LACE index can be applied to all hospitals that strive to optimize value-based medical care. This finding will help healthcare provisions and professionals to reduce 30-day readmissions by giving them insight into implementing it in the most effective strategies identified in this review.

## 6. Limitations

Our study has some limitations. First, an extensive systematic review was limited in some eligible studies that were published in English with full-text access. Second, the outcome of the predictive models was restricted to 30-day readmissions with four variables, future studies can utilize 90-day or one-year readmissions to get the exact consistency of predictions by adding the supportive additional variables. Third, meta-analyses are performed based on the heterogeneous cohort data, target population, source of data, period, and sample size at the country level. Fourth, all the reviewed studies have limited samples within the individual hospital which makes the review more open to publication bias. It was noted according to the sample size and reported in Table 1. However, certain inherent flaws are associated with it such as the selection of studies. The LACE index identifies patients at risk of readmission, but it does not necessarily enable the determination of the factors leading to readmission in specific cases. As this is the first systematic review to address the LACE index, we have no comparable systematic reviews to benchmark against. Also, this study only reviewed studies showing successful 30-day readmission risk prediction with the LACE index.

## Figures and Tables

**Table 1:** Summary of the Included Studies Regarding Lace index to predict 30-day readmissions with Quality Assessed Using the Cochrane Risk of Bias Tool.

Author (s)	Study Design/Data Source			Period	Target Population	Measurement /Analysis Model	Outcome	Risk of Bias
Caplan et al. [12]	RC	EMR	University hospital	2017–2018	Brain tumor	LACE index	30–90 day readmission	NA
Damery et al. [19]	RC	EMR	University hospital	2013–2014	All cause	LACE index	30-day readmission	Low
Dobler et al. [20]	RC	EMR	Tertiary hospital	2006–2016	Pneumonia	LACE index	30-day readmission	Low
Gruneir et al. [10]	RC	Data base	OHIP	2007	All cause	LACE index	30-day readmission	Low
Hakim et al. [21]	RC	EMR	University hospital	2006–2016	COPD	LACE index	30-day readmission	Low
Ibrahim et al. [13]	RC	EMR	University hospital	2016–2018	HF	HOSPITAL Score, LACE index, LACE+ index	30-day readmission	Moderate
Labrosciano et al. [22]	RC	Data base	NHS—PAS	2017–2019	All cause	LACE index	age-specific readmissions	Low
Linzey et al. [14]	RC	EMR	University hospital	2017	Nuro surgery	LACE index	30-day readmission	NA
Miller et al. [15]	Survey	EMR	Referral hospital	2015	All cause	LACE index	30-day readmission	Low
Robinson et al. [16]	RC	EMR	University hospital	2015–2016	All cause	LACE index, HOSPITAL score	30-day readmission	Moderate
Regmi et al. [17]	RC	EMR	University hospital	2016–2018	HF	HOSPITAL score, LACE index, and RAHF scale	30-day readmission	Moderate
Wang et al. [18]	RC	EMR	Public hospital	2011–2015	HF	LACE index	30-day readmission	Low
Yazdan et al. [11]	PC	EMR	University hospital	2012–2013	HF	LACE index	30-day readmission	Low
Low et al. [29]	RC	EMR	Public hospital	2012	COPD	LACE index	30-day readmission	Low
Tan et al. [30]	RC	EMR	Tertiary hospital	2006–2010	All cause	LACE index	30-day readmission	Moderate
Cotter et al. [31]	RC	EMR	University hospital	2010	All cause	LACE index	30-day readmission	Low

EMR = Electronic medical record; RC = Retrospective Cohort; NHI = National Health insurance; PAS = patient administrative system; OHIP = Ontario Health Insurance Plan; HF = Heart failure (includes congestive heart failure); COPD = Chronic obstructive pulmonary diseases.

**Table 2:** Baseline Characteristics of LACE Index to Predict Risk of 30-day Readmissions of selected studies.

Studies	Age (Mean)	Sex (Female, %)	30-Day Readmission			C-Stat
			Total	Yes	No	
Caplan et al. [12]	62	50	238	31	207	0.69
Damery et al. [19]	55	63	84,815	4541	84,815	0.81
Dobler et al. [20]	62	NA	4508	636	3872	0.57
Gruneir et al. [10]	65	68	26,045	8854	17,191	NA
Hakim et al. [21]	59	55	2662	847	1815	0.63
Ibrahim et al. [13]	68	63	692	189	503	0.57
Labrosciano et al. [22]	98	40	829	98	731	0.62
Linzey et al. [14]	58	51	1242	104	1138	0.62
Miller et al. [15]	65	44	359	78	281	0.68
Robinson et al. [16]	62	48	432	35	397	0.58
Regmi et al. [17]	67	60	1370	507	863	0.67
Wang et al. [18]	57	47	253	62	191	0.56
Yazdan et al. [11]	73	45	378	105	273	0.59
Low et al. [29]	67	55	5862	572	5290	0.63
Tan et al. [30]	≥21	59	143,376	15,826	111,724	0.72
Cotter et al. [31]	85	50	507	90	417	0.55

C-stat = C-statistics; NA = not applicable.

## Data Availability

The data presented in this study are available on request from the corresponding author.

## Supplementary Materials

The following supporting information can be downloaded at: https://www.mdpi.com/article/10.3390/jpm12040545/s1, Table S1: PRISMA statement; Table S2: Search strategies.
